# Genotype specific microbiome divergence of humanized APOE 2, 3, and 4 mice after microbiome standardization

**DOI:** 10.1002/alz.089264

**Published:** 2025-01-03

**Authors:** Michelle L Aries, Makayla Cook, Anna Perez, Hope Gasvoda, Ashley Gooman, Tiffany Hensley‐McBain

**Affiliations:** ^1^ McLaughlin Research Institute, Great Falls, MT USA; ^2^ Touro College of Osteopathic Medicine Montana, Great Falls, MT USA; ^3^ Touro College of Osteopathic Medicine Harlem, Harlem, NY USA; ^4^ University of Providence, Great Falls, MT USA; ^5^ Montana Center for Aging Research and Education, Great Falls, MT USA

## Abstract

**Background:**

Apolipoprotein E (ApoE) is a lipid cargo binding protein that has three variants in humans, ApoE 2, 3, and 4. The ApoE 4 allele is the greatest known genetic factor for sporadic Alzheimer’s Disease. The gut microbiome (GMB) is a key essential to health, and bacterial dysbiosis can lead to poorer outcomes for disease states and an increase in microbiota and their metabolites in the peripheral. The intersection of sex, ApoE, inflammation, and gut microbiota is incompletely understood. Previous studies in humans and humanized *APOE* mice have demonstrated *APOE*‐genotype differences in the GMB. However, the vast majority of these studies used methods unable to resolve the bacteria to the genus or species level. It remains unknown if *APOE* genotype is a driver of GMB divergence over time and how GMB changes with age and sex in the context of *APOE* genotype.

**Methods:**

In this study, humanized targeted replacement mice with either *APOE* 2, 3, or 4 (APOE‐TR) were purchased from JAX. Each APOE‐TR male was bred with one of the same two C57BL/6 sisters to standardize microbiomes across lines and monitor divergence based on *APOE* allele inheritance. Stool samples were collected from the parents at breeder set up and 6 months old and from heterozygous pups at wean, and 6 months old and will be collected every 6 months, including from the homozygous F2 generation. Stool was assessed via shallow shotgun sequencing to increase the sequencing depth and enable species and strain level taxonomic resolution.

**Results:**

The pup’s microbiome resembled each other at wean despite genotype. However, by 6 months old, GMB diverged based on sex and *APOE* genotype, with genotype‐specific alterations including changes in Lachnospiraceae (p<0.001 by PERMANOVA).

**Conclusions:**

These are the first data demonstrating GMB divergence over time driven by *APOE* genotype. Studying genotype specific changes in GMB and how specific *APOE*‐specific bacteria impact inflammatory responses can aid in understanding and potentially treating some of the underlining diseases associated ApoE 4 allele including cardiovascular disease and Alzheimer’s Disease (AD).

